# Effects of Low Temperature–Ultrasound–Papain (LTUP) Combined Treatments on Purine Removal from Pork Loin and Its Influence on Meat Quality and Nutritional Value

**DOI:** 10.3390/foods13081215

**Published:** 2024-04-16

**Authors:** Jiaojiao Yuan, Cheng Yang, Jialing Cao, Lianfu Zhang

**Affiliations:** 1 State Key Laboratory of Food Science and Technology, Jiangnan University, 1800 Lihu Avenue, Wuxi 214122, China; 6210112111@stu.jiangnan.edu.cn (J.Y.); cheng.yang@jiangnan.edu.cn (C.Y.); 7210112003@stu.jiangnan.edu.cn (J.C.); 2 College of Food, Shihezi University, Beisi Road, Shihezi 832003, China

**Keywords:** purine, red meat, gout, high uric acid, ultrasound, papain

## Abstract

A combined pretreatment method of “low temperature-ultrasound-papain” (LTUP) was proposed to remove the purine of pork loins. Compared with untreated pork loin, under optimal conditions (temperature 58 °C, ultrasound density 100 W/cm^2^, and papain concentration 0.085%), the purine removal rate of treated pork loin could reach 59.29 ± 1.39%. The meat quality of pork loin treated with the LTUP method such as hardness and chewiness decreased by 58.37% and 64.38%, respectively, and the in vitro protein digestibility was increased by 19.64%; the cooking loss was decreased by 15.45%, compared with the simulated household blanching process (HT). In view of the high purine removal rate, the losses of free amino acids and soluble peptides were acceptable and reasonable. SEM and LF-NMR results showed that low temperature and ultrasound combined with papain treatment opened a channel for purine transfer and promoted purine dissolution by affecting the protein structure of pork loin. In addition, the migration of water within the muscle tissue was also related to purine removal. In summary, LTUP is recommended as an efficient and green way for the meat industry to remove purine.

## 1. Introduction

Purine is a kind of nitrogen-containing compound with a double heterocyclic structure, which mainly includes hypoxanthine, adenine, guanine, xanthine, and their derivatives, such as nucleic acid, ATP, ADP, and so on [1]. In humans, all of them are eventually metabolized to uric acid. Uric acid can provide oxidation resistance and scavenge free radicals but excessive accumulation of uric acid in the human body will increase the risk of hyperuricemia and induce gout [2]. Moreover, foodstuff is the main external ingestion of purine [3]. Pork is rich in hypoxanthine and adenine [4], which are the main uric acid purines [5], and account for about 90% of the total purine content in pork [6]. A large intake of pork will cause a uric acid elevation in human blood [7]. A low-purine diet can reduce serum uric acid levels and relieve clinical symptoms of gout [8]. In China, pork is usually scalded briefly to remove bad smell before preparing a dish. Consideration and optimization of the pretreatment procedure to reduce the purine content need to be further explored.

Meat is rich in nutrients and has a special flavor [9] but its high purine content restricts the consumption of people with high uric acid and gout, resulting in this group of people facing problems such as dietary monotony and nutritional deficiencies [10]. Currently, there are fewer studies related to purine reduction in meat, with a single method that does not synthesize the effect of the method on meat quality [11,12]. In particular, there are even fewer studies related to purine reduction in livestock and poultry meat, which may be due to the fact that the muscle tissue of livestock and poultry meat is tightly wrapped and purine substances are not easily soluble [13,14]. Purine is a highly soluble and polar substance with stable physical properties, so it is a feasible method to reduce purine content by increasing its solubility in solvents.

Meat quality is an important indicator of consumer satisfaction with meat consumption and low-temperature cooking; ultrasound and papain all have the ability to improve meat quality. Low-temperature cooking below 60 °C is increasingly used in the food industry compared to high-temperature cooking, where pork tenderness and cooking losses are significantly improved [15]. In the food sector, ultrasonic has been widely used to assist or replace traditional processing techniques [16,17,18,19]. After ultrasonic treatment, “sonic pores” are formed on the cell surface, which can promote mass transfer [20]. Papain is commonly used for meat tenderization, which can decompose myofibril and connective tissue of muscles [21], thus increasing the myofibril gap, which can act as a channel for water transfer. 

The combination of low temperature, ultrasound, and papain may have a better purine-removing effect and can potentially improve the quality of meat but it has not been studied before. This study proposed a green and effective method for purine reduction in pork loin and analyzed the effect of this method on the quality and nutritional value of pork loin. This study not only helps to enrich the method of purine reduction in livestock and poultry meat but also provides new ideas for the dietary therapy of people with high uric acid and gout, as well as providing a reference for the production of low purine meat products in the food industry. The theoretical study of low temperature, ultrasound, and papain to reduce the purine content of pork loin, improve meat quality, and minimize the loss of nutrients can clarify the mechanism of purine leaching, meat quality improvement, low loss of nutrients, and provide a basis and guidance for the production and processing of low-purine meat products.

## 2. Materials and Methods

### 2.1. Chemicals and Materials

Hypoxanthine, adenine, guanine, and xanthine standards were purchased from Shanghai Aladdin Bio-Chem Technology Co., Ltd. (Shanghai, China) and all the standards were chromatography-grade with purity > 98%. Papain, pepsin, trypsin, and Tris-HCl buffer were purchased from Sigma-Aldrich Co. Ltd. (St. Louis, MO, USA). All other chemicals and reagents were provided by Sinopharm Chemical Reagent Co., Ltd. (Shanghai, China) and were at least of analytical grade. Acetonitrile was of chromatography grade.

### 2.2. Meat Samples

Three fresh pork loins of a boar (each weight about 350 g, longissimus thoracis, chilled at 4 °C for 24 h after slaughter) were purchased from the VANGUARD Co. Ltd. (Wuxi, China) and disposed of within one hour. Fascia and visible fat were trimmed and discarded. The pork loins were cut into 2 × 2 × 2 cm cubes weighing 9.0 ± 0.5 g. After cutting, each cube was individually sealed in separate plastic bags and allocated to different treatments.

### 2.3. Single Factor Experiment

**Figure 1:**
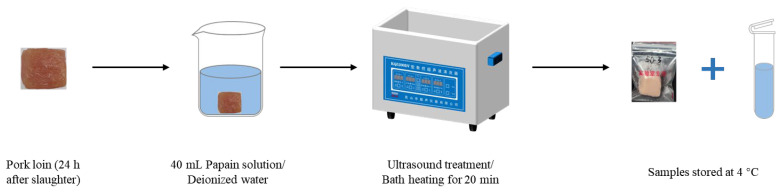
Flow diagram of treatment on pork loin.

The control variable method was used to investigate the potential impact of temperature, ultrasound, and papain on the purine removal rate of pork loin. Each cube was placed individually in a beaker containing 40 mL liquid (papain solution or deionized water). The beaker was then immediately subjected to the ultrasonic cleaning machine at a set temperature for 20 min. The treated cube was then packaged in individual plastic bags and the remaining liquid was stored at 4 °C for further analysis (Figure 1). Forty-five cubes were used in total.

#### 2.3.1. Temperature

The ultrasound density was 75 W/cm^2^. The papain concentration was 0.08%. The temperatures were set to 30 °C, 40 °C, 50 °C, 60 °C, and 70 °C.

#### 2.3.2. Papain Concentration

The ultrasound density was 75 W/cm^2^. The temperature was 60 °C. The papain concentration was set to 0.00%, 0.04%, 0.06%, 0.08%, and 0.10%.

#### 2.3.3. Ultrasound Density

The temperature was 60 °C. The papain concentration was 0.08%. The ultrasound density was set to 0 W/cm^2^, 50 W/cm^2^, 75 W/cm^2^, 100 W/cm^2^, and 125 W/cm^2^.

### 2.4. Response Surface Design of Combined Purine-Removing Processing for Pork Loin

For the single-factor experiments on the purine removal rate of pork loin, in order to maximize the promotion of purine dissolution from pork loin in a shorter period of time, an appropriate amount of solvent must be used. Response surface methodology (RSM) was employed to assess the relationship between factors and responses and the interaction among three independent factors [13]. The Box–Behnken design consisted of 17 runs (Table 1) and 51 cubes were used.

### 2.5. Evaluation of Optimal Combined Purine-Removing Processing

Through the response surface experiments described above, the process conditions that maximized the purine removal rate of pork loin were obtained. The processing was evaluated at following. Pork loin was immersed in deionized water and no ultrasonic treatment was applied as a blank control group (WT); the simulation process of the household blanching group (JT) was as follows: a beaker containing pork loin and ultrapure water was put into a water bath and heated until the water boiled and the whole process lasted for 10 min. Purine removal rate, texture profile, cooking loss, free amino acid dissolution, soluble peptide dissolution, and in vitro protein digestibility were evaluated as indicators. The optimal response surface treatment group (CT) was compared with the home blanching treatment (HT) and the blank control treatment (WT).

#### 2.5.1. Purine Removal Rate

At present, most researchers generally use high-performance liquid chromatography (HPLC) to detect purines and typically use strong acids to release purines for further detection [22]. In this experiment, the purine content in meat and water was determined by reversed-phase high-performance liquid chromatography (HPLC), according to the method of Kaneko [4] with some modifications. The standard solutions of hypoxanthine, adenine, xanthine, and guanine were prepared, respectively. Hypoxanthine and adenine were mainly detected. The sample was placed in a tube, 10% perchloric acid (PCA) was added, and it was then placed in boiling water for 60 min followed by ice-cooling. The pH of the resultant mixture was adjusted to 12.4 ± 0.1 with a potassium hydroxide solution. All the mixed liquid samples were then filtered using a 0.22 μm filter for further HPLC analysis.

A Waters Alliance 2695 liquid chromatography system (Waters, Milford, MA, USA), including a diode array detector (DAD) was used. The column was Waters AtlantisdC18 (4.6 mm × 250.0 mm × 5.0 µm) and analyzed at 25 °C. The mobile phase was 3% acetonitrile at a constant flow rate of 1.0 mL/min, the injection volume was 10 µL, and the UV absorbance was 254 nm. Each sample was detected in triplicate. The hypoxanthine and adenine content of samples were calculated by the standard curve and the purine removal rate was calculated according to the following formula:

(1) $$Purine\;removal\;rate=\frac{W_{r}}{{W_{t}+W}_{r}}\times 100\%$$

where *W_t_* is the hypoxanthine and adenine content of meat (mg/100 g), and *W_r_* is the purine content of water (mg/100 g).

#### 2.5.2. Texture Profile Analysis

After cooking loss determination, cubes were used for texture profile analysis. A texture analyzer (Stable Micro System, TA: XT2I, Godalming, UK) was used for texture profile analysis based on Pan et al. [23] with some modifications. Cooked cubes (under 60 °C, 20 min) were cut into uniform size (1 × 1 × 1 cm) after being cooled to room temperature. The texture of the cube was measured by the P/35 probe, and two compression tests were carried out with an interval of 5 s. The pre-test speed, test speed, post-test speed, and auto force were, respectively, 2.0 mm/s, 1.0 mm/s and 5.0 mm/s, 2.0 g. Hardness, springiness, cohesiveness, chewiness, and resilience were selected as the testing indexes. Values were repeated six times per sample and were averaged.

#### 2.5.3. Cooking Loss

The cooking loss was measured by the method of Inmaculada Gómez et al. [24]. Each fresh meat cube was wiped with filter paper to remove surface water and weighed as *W*_1_ before being treated. After being treated and cooled to room temperature, the surface water of it was wiped off, whose weight was recorded as *W*_2_. The cooking loss was calculated by the following equation:

(2) $$Cooking\;loss=\frac{{W_{1}-W}_{2}}{W_{1}}\times 100\%$$

#### 2.5.4. Free Amino Acid Analysis (FAA)

FAA content was based on the experimental method of Liu et al. [25]. Chloroform (10%) was added to the sample with the same volume and reaction for 1 h. The mixture was centrifuged at 12,000 rpm for 30 min and the supernatant was collected and filtered using a 0.22 μm pore filter for HPLC analysis. The Agilent 1100 HPLC system (Agilent 1100, Agilent Technology, Santa Clara, CA, USA) was used to analyze the FAA, which consisted of a quaternary pump (G1311A), autosampler (G1313A), online degassing device (G1322A), and variable wavelength detector (VWD) detector (G1314A). The Agilent Hypersil ODS (4.0 × 250 mm^2^, 5 µm; Agilent Technology, Santa Clara, CA, USA) column was used at 40 °C column temperature. The HPLC mobile phase A was 80.9 mmol/L CH_3_COONa:CH_3_CN: MeOH (1:2:2, *v*/*v*, pH = 7.2), the mobile phase B was 27.6 mmol/L CH_3_COONa: THF:(C_2_H_5_)_3_N (500:2.5:0.11, *v*/*v*, pH = 7.2), and the flow rate was 1.0 mL/min. Gradient elution: 50% B for 17 min, 100% B for 20.1 min, and 0% B for 24 min. The detection wavelength was 338 mm and proline was detected at 262 nm.

#### 2.5.5. Soluble Peptide Analysis

The content of the soluble peptide was measured according to Li et al. [26] by using a Spectra Max M5 Enzyme Labeler. Five milliliters of 15% trichloroacetic acid (TCA) were added to an equal sample volume of sample. The mixture was subjected to stand for 30 min to precipitate the protein and then centrifuged (5000 rpm for 15 min at 4 °C). The BCA protein determination kit was used to detect the absorbance of the supernatant at a wavelength of 540 nm. The content of soluble peptide was obtained by the standard curve (Y = 0.4523X + 0.0667 and R^2^ = 0.9946).

#### 2.5.6. In Vitro Protein Digestibility

The in vitro protein digestibility of cooked pork loin was carried out by Wen et al. [27] with some modifications. Briefly, chopped meat samples (2.0 g, each) were homogenized in 8 mL phosphate buffer (PBS) (0.01 M pH 7.0) for 2 × 30 s at 12,000 rpm with an interval of 30 s cooling between bursts. Then, the adjusted pH of the mixture was 2.0 ± 0.1 with 1 mol/L HCl and 2.0 mL of pepsin (≥400 units/mg of protein, from porcine gastric mucosa, product number P7125, Sigma-Aldrich, St. Louis, MO, USA) (0.48 g:15 mL 0.1 M HCL) was added. The mixture was shaken continuously at 37 °C for 2 h and 1 mol/L NaOH was used to end enzyme activity by adjusting the pH to 7.5 ± 0.1 on ice. Then, two milliliters of trypsin (1645 units/mg of protein, from porcine pancreas, lyophilized powder, type II-S, product number T7409, Sigma-Aldrich, St. Louis, MO, USA) (0.2889 g:12 mL 0.01 M pH 7.0 PBS) were added and maintained under the same conditions mentioned above. After 2 h of trypsin digestion, the mixture was heated to 95 °C for 5 min to terminate enzyme activity. The digestibility was calculated by the following equation: where DT was the digestibility, *M*_1_ is the protein content in meat after digestion, and *M*_0_ is the initial protein content in meat before digestion.

(3) $$DT=\frac{M_{0}-M_{1}}{M_{0}}\times 100\%$$

### 2.6. Scanning Electron Microscope (SEM)

The pork treated under different processing conditions was cut into 2.0 × 2.0 × 2.0 mm pieces along the myofibril direction. First, 0.2 mol/L glutaraldehyde was added and kept at 4 °C for 24 h. Then, each piece was eluted three times with a sodium phosphate buffer. After elution with 30%, 50%, 70%, 80%, 90%, and 100% ethanol solutions for 10 min each, the sample were freeze-dried. Finally, the drying pieces were sprayed with gold and observed with a scanning electron microscope (SEM) (SU8100, Hitachi High-Tech Co., Ltd., Tokyo, Japan). The settings of the scanning electron microscope were voltage 3.0 kV and magnification 4000 times.

### 2.7. Low Field Nuclear Magnetic Resonance (LF-NMR) Analysis

Referring to the method of Sun Hongxia et al. [28], the meat was wrapped with plastic wrap and placed into a glass tube with a diameter of 25 mm, inserted into an NMR analyzer, and the transverse relaxation time T_2_ of the meat was determined by using the CPMG sequence. The time interval of repeated sampling (T_W_) was set to 3000 ms, the cumulative number of scans (NS) to four, the number of echoes (NECH) to 6000, and the number of iterations to 1,000,000.

### 2.8. Statistical Analysis

Each sample was extracted in triplicate. A response surface data analysis was conducted by using the Design Expert. V8.0.6 (Minneapolis, MI, USA) software. Results were expressed as mean ± standard deviation (SD) and analyzed by one-way ANOVA and Duncan’s multiple comparisons (SPSS 26.0). Differences were considered significant when *p* < 0.05. GraphPad Prism 8.0.2 (GraphPad Software Inc., San Diego, CA, USA) was used for graphs.

## 3. Results and Discussion

### 3.1. Purine Detection

**Figure 2:**
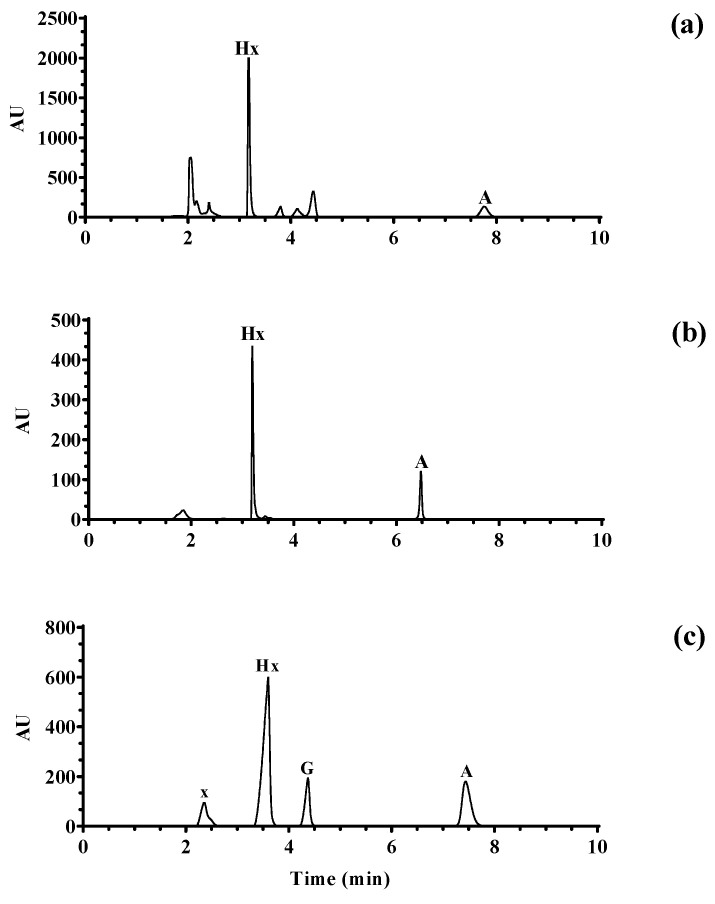
HPLC chromatogram of purines in pork loin (**a**) and water (**b**) and purine standards (**c**) obtained with constant detection wavelength at λ = 254 nm.

The mixture of the above four standard purines was detected by HPLC. Four purine standards can be separated and the retention time (xanthine: 2.358 min, hypoxanthine: 3.598 min, guanine: 4.347 min, and adenine: 7.437 min) is reasonable (Figure 2c). The chromatogram of hypoxanthine and adenine content in pork loin can be separated (Figure 2a) and the chromatogram of hypoxanthine and adenine content in broth also can be separated (Figure 2b). It can be seen that hypoxanthine peaked at around 3 min and adenine peaked at around 7 min. One must take three known purine contents of the sample solution and add different gradient mass concentrations of hypoxanthine and adenine standard solutions to it in turn; the actual value of the resulting mixture in the optimized chromatographic conditions can be used for qualitative and quantitative determination, by dividing by the spiked recovery theoretical value.

As we can see from Table 2, The fit of the two purine bases was good and the R^2^ was greater than 0.99, with a wide linear range, which can meet the detection of hypoxanthine and adenine in pork loin. The LOD was in the range of 0.10~0.13 mg/L and the LOQ was in the range of 0.30~0.45 mg/L, which indicated that the method was sensitive. The recovery of spiking was in the range of 90~110%, which indicated that the method had high accuracy and precision. The RSD was 0.05% and 0.10%, which indicated that the method had high recovery. The RSD was 0.05% and 0.10%, respectively. The spiked recoveries were in the range of 90~110%, indicating that the method has high recoveries. The RSDs were 0.05% and 0.10%, respectively, indicating that the method has good precision and high reproducibility. In conclusion, the optimized purine assay can be used for the quantitative determination of hypoxanthine and adenine in pork loin and broth.

### 3.2. Effect of Different Treatments on the Purine Removal Rate of Pork

**Figure 3:**
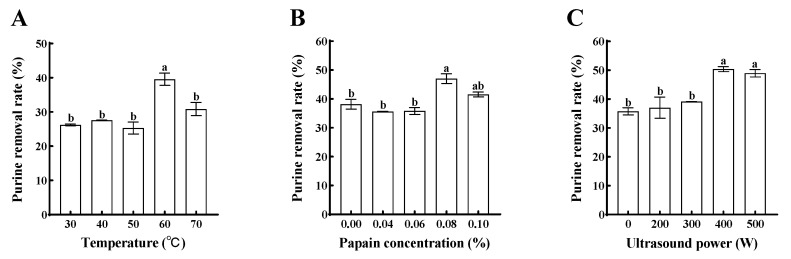
Effects of different treatments on the purine removal rate of pork loin. (**A**) Temperature, applied with 75 W/cm^2^ ultrasound and 0.08% papain concentration; (**B**), Papain concentration, applied with 75 W/cm^2^ ultrasound and bath at 60 °C; (**C**), Ultrasound density, applied with 0.08% papain concentration and bath at 60 °C. Different letters (a,b) indicate significant differences (*p* < 0.05).

The higher the purine removal rate, the better the purine-reducing effect of the treatment condition. The purine removal rate of pork loin firstly increased and then decreased when the temperature ranged from 30 °C to 70 °C and reached the maximum at 60 °C, which was 29.95% (Figure 3A, *p* < 0.05); this concentration was chosen as an intermediate level for RSM optimization. When the temperature continued to increase to 70 °C, the purine dissolution of pork loin decreased to 23.99%. It can be seen that the effect of temperature on the purine dissolution rate has two sides. High temperature can promote the dissolution of purine in pork loin but at the same time, the temperature continues to increase in the dissolution of pork purine, which showed inhibition. The reason may be due to the fact that, on the one hand, the lower rate of molecular thermal movement under low-temperature conditions is not conducive to the exchange of substances inside and outside the meat. On the other hand, when the temperature exceeds 60 °C, the spacing between myogenic fibers of the meat is significantly reduced [29,30], leading to an increase in the resistance to purine dissolution and hence a decrease in the removal rate of purines from pork loin at 70 °C. Specifically, the results of E. Tornberg [31] showed that most myoplasmic proteins aggregate between 40 °C and 60 °C but the coagulation of some of these proteins can be extended up to 90 °C. For myofibrillar proteins, the unfolding of their structure begins at 30–32 °C, followed by protein–protein binding at 36–40 °C and gelation begins at 45–50 °C. Between 53 and 63 °C, collagen begins to denature, followed by the contraction of myofibrils and possible dissolution and gelatin formation upon further heating. Thus, thermal denaturation of muscle tissue proteins may have some relevance to the dissolution of purines in pork loin.

When the concentration of papain was less than 0.06%, the purine dissolution rate of pork loin was low, while the purine dissolution rate of pork loin was significantly increased (Figure 3B, *p* < 0.05) to 47.17% with the addition of 0.08% papain. And when the concentration of papain continued to increase, the purine dissolution rate of pork loin began to decrease. This may be due to excessive hydrolysis of muscle tissue proteins by papain. Thus, a papain concentration of 0.08% was chosen as an intermediate level for RSM optimization.

As the ultrasonic power increased, the purine dissolution rate of pork loin first increased and then decreased. The increase in purine dissolution rate was not significant (Figure 3C, *p* < 0.05) at an ultrasonic power of less than 75 W/cm^2^ and the purine dissolution rate of pork loin increased significantly (*p* < 0.05) at 100 W/cm^2^, which was chosen as an intermediate level for RSM optimization. The purine dissolution rate of pork loin decreased at 125 W/cm^2^ compared with that of pork loin at 100 W/cm^2^. The purine dissolution rate of pork loin decreased at 500 W compared with that of pork loin at 125 W/cm^2^. Compared with 100 W/cm^2^, the purine removal rate of pork loin at 125 W/cm^2^ decreased. This may be due to the fact that the high-intensity ultrasound caused excessive fragmentation of proteins in the muscle tissue and blocked them in the interstitial space of myofibrils, which hindered the removal of purines.

### 3.3. Optimization of the Purine Removal Process of Pork Loin by RSM

**Figure 4:**
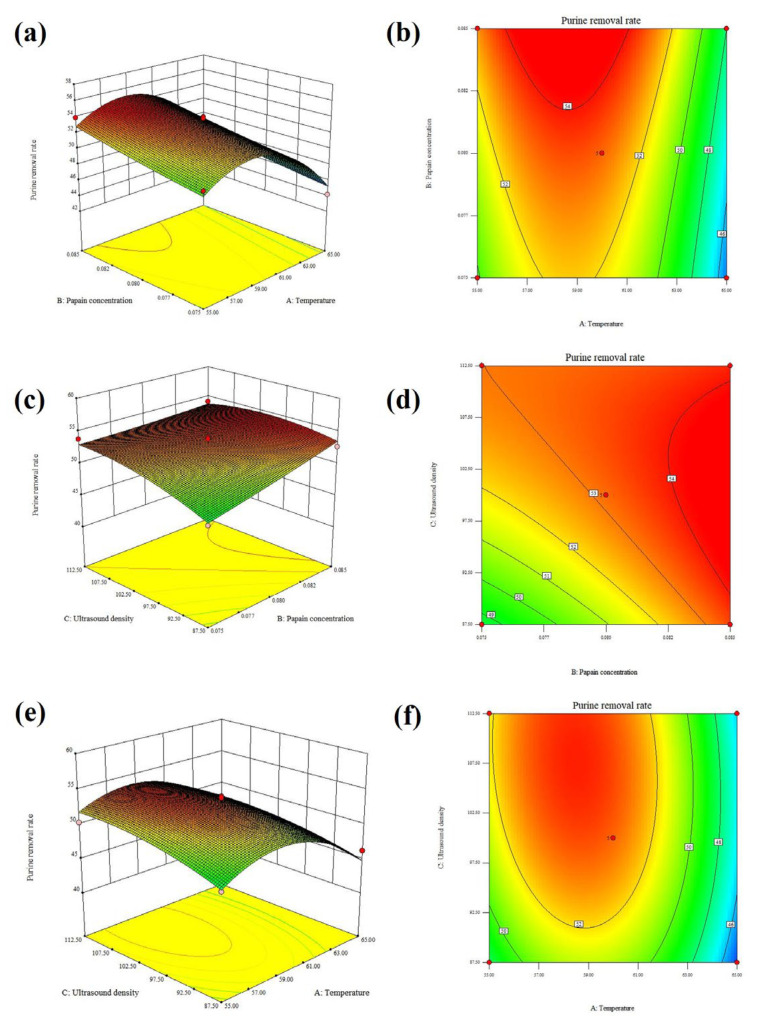
The 3D response surface plots (**a**,**c**,**e**) and 2D contour plots (**b**,**d**,**f**) of interactions between the temperature, the papain concentration, and the ultrasound density on the purine removal rate of pork loin.

To further optimize the combined purine removal process of pork loin, using purine removal rate as the indicator, the Box–Behnken response surface was used to perform three factors and three levels of temperature (A), papain concentration (B), and ultrasound density (C). The *p*-value (<0.05) was significant, the correlation coefficient and lack of fit items were insignificant, and the linear items A, B, and C were significant (Table 3, *p* < 0.05), indicating that the RSM test was meaningful. The interaction among fixed main factors (temperature, papain concentration, and ultrasound density) was not significant. The R^2^ was 0.9324, showing that only 6.76% of the variance could not be explained by the model and that the fit was good. The Adj-R^2^ was 0.8454, a highly adequate reflection of the model. And the R^2^ was close to the Adj-R^2^, indicating that the model can accurately reflect the effects of the above three factors on the purine removal rate of pork loin. The following equation between the purine removal rate (Y) of pork loin and temperature (A), papain concentration (B), and ultrasound density (C) was Y = 53.16 − 2.35A + 1.42B + 1.15C − 0.058AB − 0.51AC − 1.03BC − 4.30A^2^ + 0.14B^2^ − 1.06C^2^. The 3D response surface plots and 2D contour plots were the graphical representation of this regression equation (Figure 4).

The RSM results showed that under the predicted optimal treatment conditions of 58.56 °C, 106.61 W/cm^2^, and 0.085% papain, the predicted value of the purine removal rate was 55.08%. Combined with the actual operation, the optimal process conditions were adjusted to 58 °C, 100 W/cm^2^, and 0.085% papain concentration. It was verified three times that the purine removal rate of pork loin under this condition was 59.29 ± 1.39%, which was very close to the value predicted by the model, indicating that this model was accurate and satisfactory.

### 3.4. Purine Removal Rate Analysis

**Figure 5:**
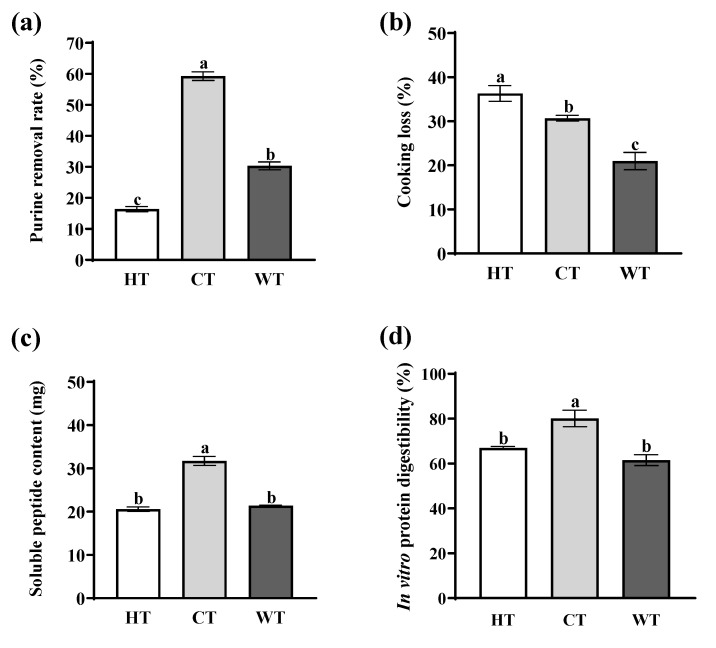
Purine removal rate (**a**), cooking loss (**b**), soluble peptide content (**c**), and in vitro protein digestibility (**d**) of pork loin under different treatments. HT: the household blanching process; CT: the combined treatment with 58 °C, 0.085% papain concentration, and 100 W/cm^2^ ultrasound density; WT: the water treatment. Different letters (a–c) indicate significant differences (*p* < 0.05). The error bar represents the standard error of the mean.

Compared with HT and WT, CT had the highest purine removal rate (Figure 5a, *p* < 0.05), which reached 59.29 ± 1.39% and increased by 72.33% and 48.87%, respectively. HT has the lowest purine removal rate. Under short stewing, a high-temperature water bath resulted in a drastic denaturation of proteins on the surface of the meat and shrank the space of fibers more quickly, closing the water channel between the myofiber gap. On the one hand, ultrasound and papain may promote the migration of water with dissolved purines. On the other hand, the molecular structure of DNA and ATP contains adenine bases. According to ALFRED et al. [32], DNA damage can be caused by ultrasonic cavitation and mechanical acoustic chemistry, resulting in the breaks in DNA and the release of purine. Furthermore, Yoon et al. [33] have found that ultrasound triggered the opening of connexin 43 hemichannels on the plasma membrane, releasing ATP into the extracellular space. Wang et al. [34] found that the mushroom DNA was significantly degraded when the temperature exceeded 80 °C.

### 3.5. Texture Profile Analysis

Texture is an important method for assessing the quality of meat products and affects consumer acceptance and recognition [23]. In this study, five textural parameters that can be used to assess meat quality were identified, namely, hardness, elasticity, cohesion, chewiness, and reparability. There was a significant difference among hardness, springiness, cohesiveness, chewiness, and resilience of CT compared to those of JT (Table 4, *p* < 0.05). CT had lower hardness and chewiness, with CT having 54.35% and 60.65% less hardness and chewability, respectively, compared to JT. Collagen provides a hard texture to the muscle and collagen breakdown leads to the destruction of muscle fibers and softening of the meat [35]. The muscle collagen begins to denature at 65 °C and the toughness of the muscle increases as the heating temperature rises [15]. More severe shrinkage and toughening of the meat occurs when the temperature is 100 °C [36], which explains the highest hardness of the meat pieces under the conventional blanching group treatment at home. CT had better hardness and chewiness and more resilience and cohesiveness compared to WT without the application of ultrasound and papain, which may be due to the tenderizing effect of ultrasound or papain [37].

### 3.6. Cooking Loss Analysis

Cooking loss refers to the loss of moisture and soluble substances during the cooking process of meat [38]. A high cooking loss will reduce the juiciness of the meat and affect its eating quality [39]. The cooking loss of HT was highest, compared with CT and WT (Figure 5b, *p* < 0.05). Wang et al. [40] results also showed that the cooking loss of muscles at 100 °C was significantly higher than that at 60 °C and the cooking loss increased with the increase in temperature under the same cooking time. At 55–65 °C, the sarcoplasmic protein in muscle was denatured and the thermal contraction of the collagen fibers forced a large amount of free water in muscle fiber cells to flow out. At 60–100 °C, the muscle protein was severely denatured, free water was further lost, and soluble collagen was further thermally degraded to form gelatin dissolution [41]. Compared with WT, the high cooking loss of CT may be due to the high-intensity sonication, which weakened the bond between muscle proteins and water, resulting in a water transfer [42]. In addition, papain can reduce the water-holding capacity [43] and affect the cooking loss of muscle [44]. In the current research, papain concentration can lead to greater cooking losses [44].

### 3.7. Free Amino Acid Dissolution Analysis

Free amino acids are an essential parameter for evaluating the nutritional value of pork [45]. The experiment aimed to measure the nutritional values of LTUP treatment on purine-removing treatment. As can be seen from Table 5, the loss of the total free amino acids content in CT was higher than that of HT and WT. It showed the same trend of the purine removal rate and the soluble peptide content. The Glu, Gly, Arg, Met, Phe, Leu, and Lys were almost 2–4 times higher than those of HT and WT, which showed that papain has worked. According to Ha et al. [46], papain is a cysteine protease and has broad-spectrum activity, which is efficient in hydrolyzing actomyosin, titin, and nebulin. Herranz et al. [47] have added papain to sausages to increase the amount of free amino acids. Furthermore, some results suggested that the amount of certain amino acids has been significantly increased in ultrasound-treated meat samples and ultrasonic had an impact on the breaking down of low molecular weight polypeptides or individual amino acids.

### 3.8. Soluble Peptides Dissolution Analysis

The soluble peptides include small peptides (MW < 10 kDa), free amino acids, etc., which can reflect the degree of hydrolysis of muscle proteins [35]. The soluble peptide content in the CT solvent was significantly higher than that in the other two groups (Figure 5c, *p* < 0.05), about 2.25 times that in the JT group and 2.17 times that in the WT group, which was in agreement with the results of the determination of free amino acids. This indicates that the loss of soluble peptides was exacerbated by low temperature and ultrasound combined with papain treatment. The breakdown of proteins into peptides by papain could explain these results.

### 3.9. In Vitro Protein Digestibility Analysis

In vitro protein digestibility reflects how much protein in food is hydrolyzed by pepsin and trypsin in the digestive tract [48]. CT has the highest in vitro protein digestibility, which increased by 19.64% and 30.16%, respectively, compared with HT and WT. This result showed that LTUP treatment improved the in vitro protein digestibility of pork loin (Figure 5d, *p* < 0.05). According to Jiang et al. [49], ultrasound can enhance the hydrophobicity of protein on the surface of pork loin, exposing more hydrophobic amino acid residues and making it easier to digest by proteases. Similar related studies have also shown that high-intensity ultrasound treatment improved the in vitro protein digestibility of meat [37].

### 3.10. Microstructure Analysis

**Figure 6:**
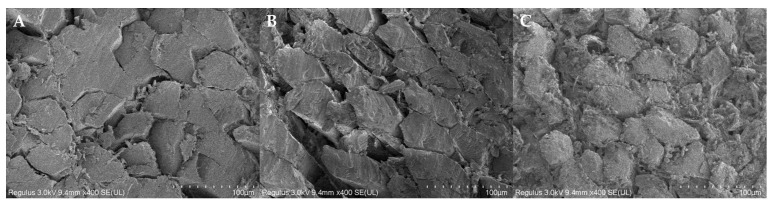
Scanning electron microscope (SEM) of pork after different treatments. (**A**): WT; (**B**): CT; and (**C**): HT. WT: the water treatment. Different letters; CT: the combined treatment with 58 °C, 0.085% papain concentration, and 400 W ultrasound density; HT: the household blanching process.

The appearance of fiber microstructure was observed in a cross-section. Under the WT treatment, the pork tissue was less damaged and the fiber structure was tidy and smooth (Figure 6A). In contrast, the fiber of HT showed that the surface was rough and the intact structure of the endomysium was severely damaged; part of the collagen was granular dissolved or blocked in the interstitial space of myofibrils, causing a smaller gap (Figure 6C). Pork treated under CT showed a loose arrangement with the largest gap and less structural damage, which can explain the high purine removal rate of CT (Figure 6B). Xiong et al. [50] also found significant myofibril gaps under combined treatment of papain and ultrasound. In addition, the interstitial space of myofibrils can act as a channel for water migration between the muscle and the environment; the looser its arrangement, the more conducive it is to the dissolution of endosomes in muscle [40]. In summary, high purine is related to fiber gap and a larger fibers gap showed a high purine removal rate.

### 3.11. Water Distribution of Pork Loin by LF-NMR

Low-field NMR techniques are often used to determine moisture in meat [51]. Water in meat can be broadly categorized into three types: bound water, immobile water, and free water. Water that is tightly bound to proteins through hydrogen bonding is bound water, which is highly stable; water that is retained in the muscle tissue due to spatial effects, etc., is not easy to flow water, which is released when the structure of the myogenic fibers changes; and the part of the water that is highly mobile and has the weakest bonding force with the muscle is free water [52]. The lateral relaxation time T_2_ is different for different types of water. The longer the relaxation time, the more free water is indicated. The distribution of transverse relaxation times of bound water, immobile water, and free water in pork loin are T_21_, T_22_, and T_23_, respectively.

**Figure 7:**
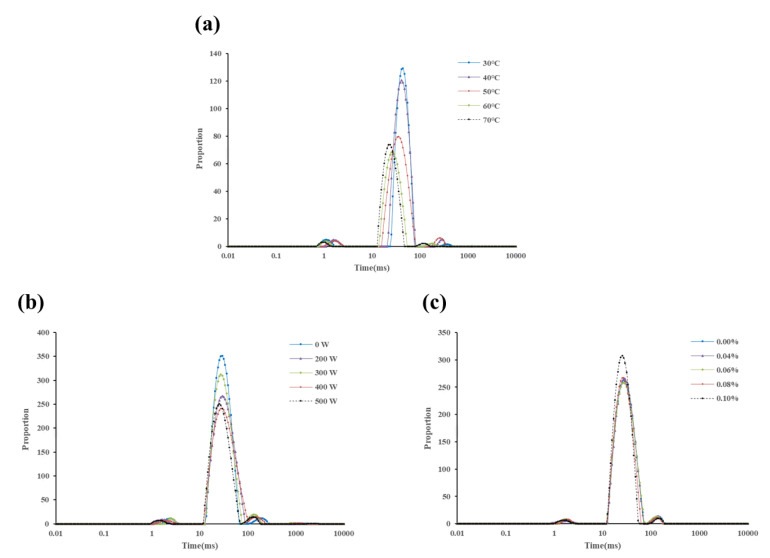
Distribution of water transverse relaxation time in pork loin under different temperatures (**a**), papain concentrations (**b**), and ultrasound densities (**c**).

The pork loin showed three proton components after treatment at different temperatures, which were T_21_, T_22_, and T_23_ (Figure 7a). The relaxation times of the three protons at different treatment temperatures were different and the higher the treatment temperature was, the shorter the relaxation time was, which was in agreement with the results of Bertram et al. [53]. It shows that the degree of freedom of the protons increases as the temperature increases. The total moisture content of pork loin was significantly lower (*p* < 0.05) at 60 °C compared to the total moisture content of pork loin after treatment below 60 °C and lower than 70 °C, which is consistent with the results of the water-holding capacity and also indicates that the dissolution of purines in pork loin is closely related to its moisture content.

It can be seen that there was no effect of different concentrations of papain treatment on the transverse relaxation time of the three protons (Figure 7b). The total moisture content in pork loin under 0.08% papain treatment was significantly lower (*p* < 0.05) than in the other treatment groups, suggesting that purine leaching was related to moisture content.

Similarly, different ultrasound density treatments had no effect on the lateral relaxation time of the three protons (Figure 7c). The total water content in pork loin decreased significantly (*p* < 0.05) with increasing ultrasound density. The total water content in pork loin at 400 W was 4622.79 ± 11.63, which was slightly higher than that at 125 W/cm^2^ (*p* < 0.05). The purine removal rate in pork loin at 500 W was lower than that at 100 W/cm^2^, which may be attributed to the protein denaturation and degradation affecting the dissolution of purines under 125 W/cm^2^.

## 4. Conclusions

The combination of low temperature, ultrasonic, and papain (LTUP) for the purine reduction in pork was explored. Under the optimal condition, temperature 58 °C, ultrasound density 100 W/cm^2^, papain concentration 0.085%, the purine removal rate can reach 59.29%. The degradation and denaturation of proteins in muscle tissues of pork loin after combined treatment with ultrasound and papain showed a correlation with high purine dissolution rate. Meanwhile, when the purine dissolution rate was high, the pork loin had lower water holding capacity, lower water content, and larger interstitial spaces in the myogenic fibers, showing a correlation between water migration and purine dissolution. In addition, LTUP significantly improved the eating quality of pork, including hardness, chewiness, and in vitro protein digestibility. Meanwhile, the cooking loss of LTUP is low and the losses of soluble peptides and amino acids were acceptable, compared with the effect of purine-reduction. Thus, LTUP has a potential application in meat products industries and can produce low purine content meat for people with high uric acid and gout.

## Figures and Tables

**Table 1:** The factors and levels for the response surface methodology design.

Factors	Levels		
	−1	0	1
Temperature-A (°C)	55	60	65
Papain concentration-B (%)	0.075	0.08	0.085
Ultrasound density-C (W/cm^2^)	87.5	100	112.5

**Table 2:** Calibration curve and validation parameters.

Purine	Test Range (mg/L)	Calibration Curve	R^2^	LOD	LOQ	Recoveries (%)	RSD
				(mg/L)	(mg/L)		(%)
Hypoxanthine	1–300	Y = 61771X + 362582	0.9916	0.1	0.32	93.44%	0.05
Adenine	1–100	Y = 75559X + 92210	0.9996	0.13	0.44	106.68%	0.1

**Table 3:** ANOVA for response surface quadratic model. a Model factors: A = Temperature (°C), B = Papain concentration (%), C = Ultrasound density (W). df ^b^: degree of freedom.

Source	Sum of Squares	df ^b^	Mean Square	F Value	*p*-Value
Model	161.18	9	17.94	10.72	0.0025
A	44.37	1	44.37	26.52	0.0013
B	16.13	1	16.13	9.64	0.0172
C	10.63	1	10.63	6.35	0.0398
AB	0.013	1	0.013	0.008	0.9316
AC	1.03	1	1.03	0.62	0.4583
BC	4.22	1	4.22	2.52	0.1561
A^2^	77.95	1	77.95	46.59	0.0002
B^2^	0.085	1	0.085	0.051	0.8279
C^2^	4.76	1	4.76	2.84	0.1357
Residual	11.71	7	1.67		
Lack of Fit	9.43	3	3.14	5.51	0.0664
Pure Error	2.28	4	0.57		
Cor Total	173.18	16			
R^2^	0.9324				
R^2^_adj_	0.8454				

**Table 4:** Changes in textural parameters of pork loin under different treatments. Dissimilar letters (a,b)—indicate significant differences (*p* < 0.05) in between-group variation. Data are presented as the mean ± standard error.

Groups	Hardness (g)	Springiness (mm)	Cohesiveness	Chewiness (g)	Resilience
HT	2405.23 ± 119.80 ^a^	0.53 ± 0.01 ^a^	0.58 ± 0.03 ^a^	809.04 ± 17.14 ^a^	0.33 ± 0.02 ^a^
CT	1001.21 ± 190.89 ^b^	0.40 ± 0.02 ^b^	0.55 ± 0.02 ^a^	288.15 ± 63.78 ^b^	0.25 ± 0.02 ^b^
WT	1097.97 ± 61.48 ^b^	0.39 ± 0.02 ^b^	0.44 ± 0.03 ^b^	318.34 ± 33.45 ^b^	0.31 ± 0.01 ^ab^

**Table 5:** Content changes of free amino acids in solvents under different treatments. Dissimilar letters (a,b) indicate significant differences (*p* < 0.05) in between-group variation. Data are presented as the mean ± standard error.

FAA	Contents (mg/mL)		
	HT	CT	WT
Asp	0.005 ± 0.003 ^a^	0.009 ± 0.002 ^a^	0.005 ± 0.003 ^a^
Glu	0.015 ± 0.005 ^a^	0.028 ± 0.002 ^a^	0.014 ± 0.003 ^a^
Ser	0.003 ± 0.000 ^a^	0.005 ± 0.003 ^a^	0.003 ± 0.001 ^a^
His	0.007 ± 0.001 ^a^	0.010 ± 0.000 ^a^	0.003 ± 0.000 ^b^
Gly	0.009 ± 0.002 ^b^	0.016 ± 0.002 ^a^	0.007 ± 0.000 ^b^
Thr	0.010 ± 0.002 ^ab^	0.014 ± 0.002 ^a^	0.005 ± 0.001 ^b^
Arg	0.008 ± 0.003 ^ab^	0.022 ± 0.001 ^a^	0.008 ± 0.002 ^b^
Ala	0.179 ± 0.041 ^a^	0.172 ± 0.040 ^a^	0.135 ± 0.018 ^a^
Tyr	0.023 ± 0.006 ^a^	0.027 ± 0.002 ^a^	0.019 ± 0.004 ^a^
Cys-s	0.003 ± 0.001 ^a^	0.003 ± 0.001 ^a^	0.002 ± 0.001 ^a^
Val	0.007 ± 0.001 ^a^	0.009 ± 0.001 ^a^	0.007 ± 0.001 ^a^
Met	0.006 ± 0.002 ^b^	0.019 ± 0.000 ^a^	0.006 ± 0.003 ^b^
Phe	0.004 ± 0.003 ^a^	0.013 ± 0.003 ^a^	0.004 ± 0.001 ^a^
Ile	0.003 ± 0.002 ^a^	0.005 ± 0.003 ^a^	0.003 ± 0.001 ^a^
Leu	0.008 ± 0.003 ^b^	0.029 ± 0.004 ^a^	0.008 ± 0.001 ^b^
Lys	0.005 ± 0.006 ^a^	0.019 ± 0.001 ^a^	0.005 ± 0.001 ^a^
Pro	0.005 ± 0.000 ^a^	0.004 ± 0.000 ^a^	0.005 ± 0.001 ^a^
Total	0.298 ± 0.058 ^a^	0.402 ± 0.0.16 ^a^	0.235 ± 0.002 ^a^

## Data Availability

The original contributions presented in the study are included in the article, further inquiries can be directed to the corresponding author.
